# 3D conformal bandpass millimeter-wave frequency selective surface with improved fields of view

**DOI:** 10.1038/s41598-021-91218-y

**Published:** 2021-06-18

**Authors:** H. Fernández Álvarez, Darren A. Cadman, Athanasios Goulas, M. E. de Cos Gómez, Daniel S. Engstrøm, J. C. Vardaxoglou, Shiyu Zhang

**Affiliations:** 1 Department of Electrical Engineering, University of Oviedo, 33203 Gijón, Spain; 2 Wolfson School of Mechanical, Electrical and Manufacturing Engineering, Loughborough University, Loughborough, Leicestershire, LE11 3TU UK

**Keywords:** Electrical and electronic engineering, Metamaterials, Applied physics

## Abstract

Conventional planar frequency selective surfaces (FSSs) are characterized in the far-field region and they are sensitive to the incidence angle of impinging waves. In this paper, a spherical dome FSS is presented, aiming to provide improved angular stable bandpass filtering performance as compared to its planar counterpart when the FSS is placed in the near-field region of an antenna source. A comparison between the conformal FSS and a finite planar FSS is presented through simulations at the frequency range between 26 to 40 GHz in order to demonstrate the advantages of utilizing the conformal FSS in the near-field. The conformal FSS is 3D printed and copper electroplated, which leads to a low-cost and lightweight bandpass filter array. Placing it in the near-field region of a primary antenna can be used as radomes to realize compact high-performance mm-wave systems. The comparison between simulated and measured conformal FSS results is in good agreement. The challenges that arise when designing, manufacturing, and measuring this type of structure are reported and guidelines to overcome these are presented.

## Introduction

Frequency selective surfaces (FSSs) have widely been studied in the literature over the years^1–5^. They are periodic resonant structures that selectively allow or prevent the transmission of electromagnetic waves. They are used as special low-pass, high-pass, bandstop, and bandpass filters^6^. The most common configurations comprise arrays of dipoles or their complementary structure (slots) arranged in a 2D planar surface. At their resonant frequency, the dipole array is characterized by totally reflecting the incident EM wave impinging on it and the slot arrays allow the transmission of it. They are used in many EM applications, such as radomes^7–10^, reflectors^11–13^, polarizers^14,15^, electromagnetic shielding^16–19^, among others.

The conventional planar FSSs are designed to be uniformly illuminated by a plane wave and are characterized in the far-field region of both transmitter and receiver antennas. However, when FSSs are used together with antennas, they are preferable to be placed close to the antennas to reduce the volume of the whole system. This means the FSSs could be in the near-field region and plane wave incidence is no longer applied. Although the antennas can be designed to have uniform near-fields that are similar to a plane wave, conformal FSSs are more versatile for antennas with different wavefronts. Moreover, it is increasingly pertinent that modern radio frequency (RF) systems use structurally integrated components including radomes and FSSs to provide both electromagnetic and structural advantages. The radomes and FSSs encompassing antennas for electromagnetic and/or environmental shielding are required to fit on a system that has a predetermined shape, due to aerodynamic and/or structural constraints. However, apart from studies on finite planar FSS in the near-field^20,21^, no rigorous study has been conducted on conformal FSS structures in the near-field region to date.

Planar FSSs are inherently sensitive to the impinging wave angles. The resonant frequency shifts and the filtering ability degrades when the angle of incidence increases. When the FSSs are used for radomes and shielding applications, they are required to provide a wide field of view (FOV) to ensure their angular stable filtering abilities, due to the arbitrary impinging wave angles in practical applications. The recent developments on the 3D FSS elements have shown some superior performance features compared with the 2D (planar) FSS elements^22,23^. One of the advantages of using the 3D elements is to enable the FSS insensitive to the incidence angle of impinging waves, and therefore improve the FOV^24–27^. However, these 3D elements increase the FSS size and weight and when combining with an antenna the resulting system is not compact at all, as the FSS is designed to work in the far-field region. A wide FOV is still a challenge for finite thin FSSs.

The development of conformal FSSs mainly faces three challenges. Firstly, the planar FSS elements suffer from dimensional distortions as they are projected on a curved surface. The shielding and filtering ability would be degraded due to deformed FSS elements. The second obstacle is the numerical solution of the problem. The analysis methods for nonplanar geometries have been presented in^28–31^, but most commercially available EM simulators experiment with different problems when dealing with numerical simulations of finite structures, especially the ones regarding the analysis of their scattering properties. Last but not least issue is related to the manufacturing of the conformal FSSs. Flexible textile-based FSSs have been reported in^32,33^, but a cut-and-paste process is usually required to wrap a thin planar FSS on a doubly curved surface (e.g. hemispheres or domes), which also leads to dimensional distortions^34^. A bandstop conformal FSS that was realized by using screen printing (with a conformal mask) to deposit the conductive patterns on a 3D printed conformal dielectric base layer was reported in^35^, but the resonant frequency of the FSS was shifted and the stop band bandwidth enlarged when compared with its electromagnetically simulated infinite planar counterpart. This was due to the distorted FSS elements on the curved surface and the oblique incident angles. Furthermore, it is well known that manufacturing metasurfaces in the mm-wave frequency band is a challenge and even more when curved FSS is required. It also worth noting that so far most authors have presented bandstop FSSs or bandpass ones that need a supporting dielectric layer^3,23,24^.

With the recent developments of additive manufacturing (AM) technology and the emergence of higher resolution 3D printers, it is possible to manufacture non-distorted element arrays on arbitrarily curved surfaces. In this paper, a fully metalized conformal bandpass FSS is proposed for the first time and compared with their planar counterpart, aiming to provide a wide field of view (FOV) but similar filtering performance including resonant frequency and the bandpass bandwidth when the FSS is placed in a near-field region. As a proof of concept, a spherical dome bandpass FSS that is illuminated by an open-ended waveguide (OEWG) feeding is presented at the frequency range between 26 and 40 GHz. It is additively manufactured on a plastic PLA former using the low-cost Fused Filament Fabrication (FFF) method that is metalized using copper electroplating. The manufacturing process including the FFF parameter and copper electroplating are optimized to ensure minimum dimensional distortion and high conductivity. The bandpass performance of the fabricated FFS was experimentally measured and compared with the simulated predictions.

## Conformal FSS

### Spherical dome conformal FSS

**Figure. 1:**
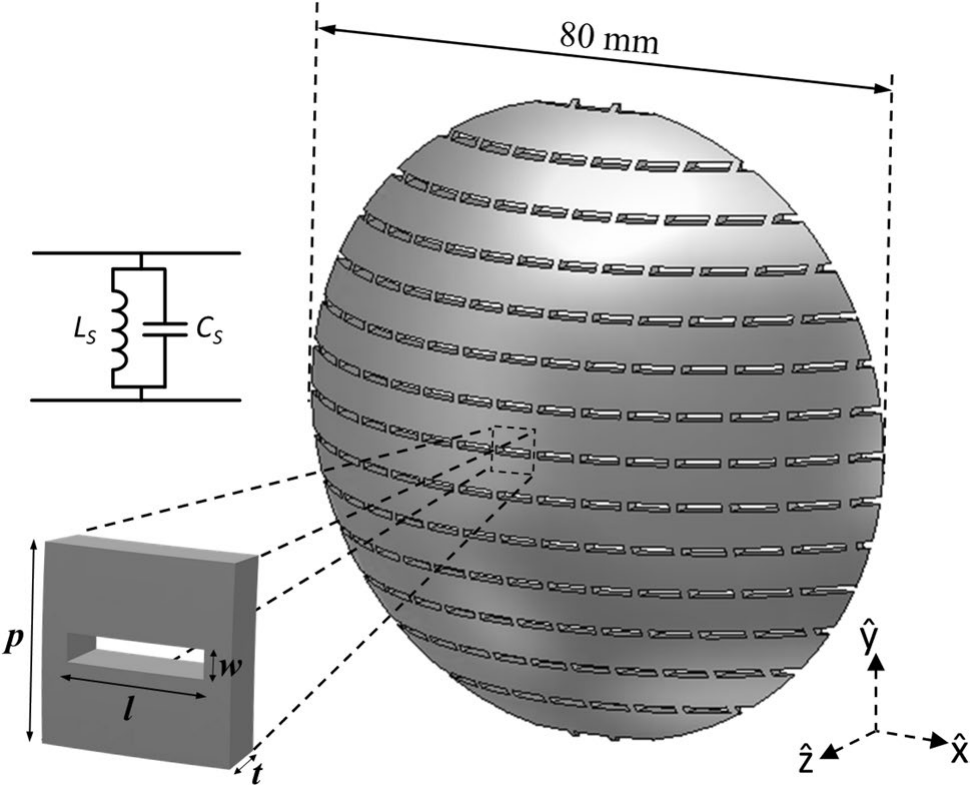
3D view of the conformal FSS, the conformally bent unit cell that are normal to the z-direction, and the equivalent circuit of the unit cell.

The FSS slot elements are placed on a spherical dome surface that has a spherical radius of 50 mm, dome height of 20 mm with a 2 mm thickness. The circular aperture diameter is 80 mm. The slots are designed as arcs in the sphere for having the radial periodicity and dimensions equal to the planar unit cell. The spherical dome conformal FSS with arc-shaped slots is shown in Fig. 1. The FSS slot is designed to work on the Ka-band with a resonance frequency at ~ 33 GHz. The following parameters are chosen: *p* = 6 mm, *l* = 4.57 mm and *w* = 0.87 mm. It should be mentioned that this takes into account the deformation that the planar slots are subjected to when projected on the sphere, contrary to the methods presented in^36^. In this work, the slots are made directly on the sphere using radial arcs instead of projecting the planar dipole slot structure into a sphere, so that the radial length of the slots can be directly controlled. The dome shape is widely used in antenna radomes. An open-ended waveguide is used to generate quasi-spherical wavefronts, and this conformal shape is able to minimize the difference in incident angles. The unit cell can be modeled as a parallel *LC* circuit as shown in the inset of Fig. 1, where the capacitor (*C*_*S*_) is due to the slot’s gap (*w*) and the inductance (*L*_*S*_) can be attributable to the metallization part of the unit cell at both sides of the slot.

**Figure. 2:**
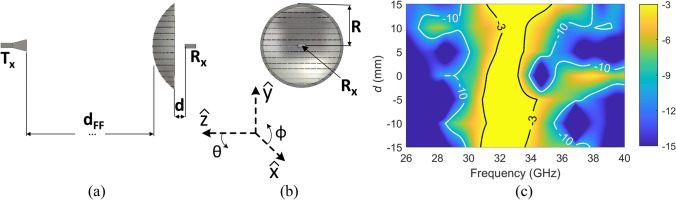
**(a)** Side view (YZ plane) of the conformal FSS; **(b)** view from the receiver (XY plane); and **(c)** simulated S21 of the conformal FSS as a function of distance and frequency, with contour lines indicates the -3 dB and -10 dB transmission bandwidth.

The full-wave simulation set-up for the conformal FSS is presented in Fig. 2(a) and (b). It consists of an OEWG WR-28 that acts as the receiver, and a Ka-band horn antenna as the transmitter^37^, which is placed at a far-field distance from the FSS. Moreover, an additional simulation, considering the same set-up (same distance between OEWG and horn), except the FSS which is removed, is simulated for calibration purposes, so the transmitted signal when the FSS is presented and when it is not are compared. It should be noticed that the approximation of using a unit cell with periodic boundary conditions and Floquet Wave excitation cannot be applied to analyze the behavior of the proposed FSS, as it has a curved shape. Moreover, it is known that full-wave simulations of planar finite FSSs require heavy computational resources, which even increase when considering curved structures due to the number of meshing elements needed to cover the conformal structure. Therefore, selecting the appropriate full-wave simulation solver is crucial for saving time while maintaining accuracy. The Time Domain Solver provided by CST Studio Suite was used in this work, taking the advantages of the incorporated Perfect Boundary Approximation and the Thin Sheet Technique to provide a balance between the affordable computational resources and accurate EM analysis, comparing with the Frequency Domain Solver and the Integral Equation Solver. Moreover, as the structure is symmetric in the XZ plane, an electric symmetry condition was established in this plane, which imposes a null electric tangential component for reducing the computational resource consumption.

Using the aforementioned configurations, the transmission through the curved FSS can be calculated. Two cases can be identified depending on the position of the OEWG regarding the FSS: when the OEWG is “inside” (negative *d* values) or “outside” (positive *d* values) the FSS. Note, the distance between the *Tx* and *Rx* is not changed in the simulation, only the FSS is moved along the *z*-axis, i.e. with variable *d*_*FF*_ and *d*. The simulated S21 results as a function of distance and frequency is shown in Fig. 2(c). The contour lines highlight the region of the − 3 dB and − 10 dB passband. From these two cases, several aspects can be highlighted: as long as the OEWG is “inside” the FSS, the FSS behaviours are similar to the infinite FSS, with the center frequency between 32 and 33 GHz and with the bandwidth of ~ 10%. This phenomenon can be explained by the ray theory. More precisely, the rays that leave the OEWG are closed to the spherical wavefront (as OEWG exhibits low directivity) and hence, impinge the FSS at similar incident angles as long as the OEWG is inside the FSS. This effect can be noticed in Fig. 2(c) or in a more detailed way in the first part of Table 1 in which the beginning, center, and end frequencies of the − 3 dB transmission passband below maximum together with the absolute and relative bandwidths are presented. On the other hand, when the waveguide is “outside” the FSS (*d* > 0 mm), the transmission band is slightly shifted to a greater extent. In this latter case, not only the transmission properties of the FSS, but also the diffraction from the edges and the spill-over losses affect the passband properties of the FSS. Indeed, as one moves away from the FSS, the EM wave emanating from the OEWG impinges the FSS with a lesser range of incidence angles. A comparison of simulation of an infinite periodic planar array of this unit cell in free space at normal incidence using periodic boundary conditions and Floquet Wave excitation was carried out and the results are included in Table 1. It shows that when the OEWG is ‘inside’ the FSS, the conformal FSS provides a similar level of passband bandwidth (~ 10%) centered at 32.6 GHz as the infinite periodic array in the far-field.

### Finite planar spherical FSS

**Figure. 3:**
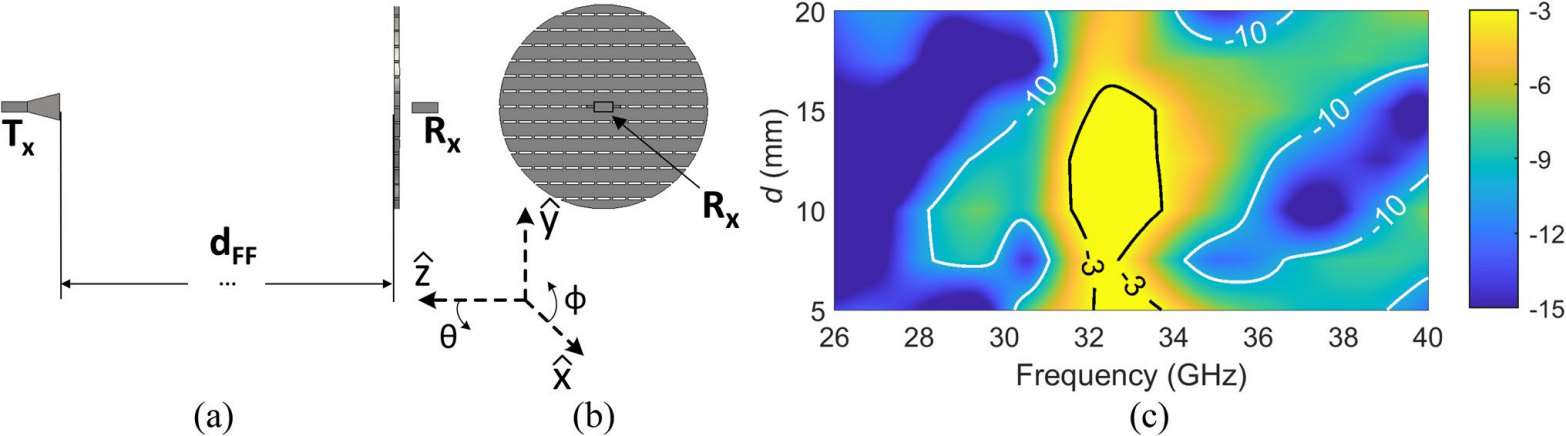
**(a)** Side view (YZ plane) of the finite planar FSS; **(b)** view from the receiver (XY plane); and **(c)** simulated S21 of the finite planar FSS as a function of distance and frequency, with contour lines indicates the -3 dB and -10 dB transmission bandwidth.

For comparison, simulations for a circular planar FSS was carried out. The simulation set-up configuration for the planar FSS is shown in Fig. 3(a) and (b). The planar FSS had the same diameter (80 mm) as the conformal FSS. Since the Rx can only be placed “outside” the FSS, the distance *d* is in positive values, ranged from 5 to 20 mm. The simulated transmission behavior is shown in Fig. 3(c) and the key results are summarized in Table 2.

**Figure. 4:**
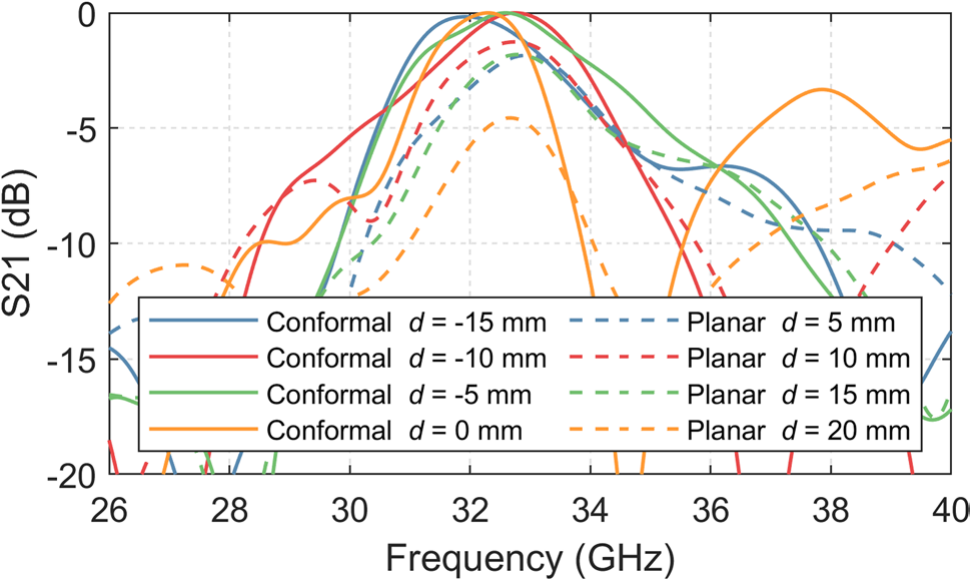
Simulated bandpass performance of the conformal FSS and the finite planar FSS with different d values.

The results indicate that planar finite FSS does not offer the same level of passband bandwidth as the infinite FSS with normal incidence, because the transmitted wave impinging the FSS from the OEWG with a smaller range of incidence angles. By contrast, it is clearly shown that the conformal FSS offers a similar passband bandwidth as the infinite FSS, which is desirable for implementing the conformal FSS in the near-field region setup. Figure 4 presents the comparison between selective simulated S21 results of the conformal and planar FSSs, which shows the conformal FSS provides a more stable bandpass bandwidth for different *d* values.

### Fields of view

**Figure. 5:**
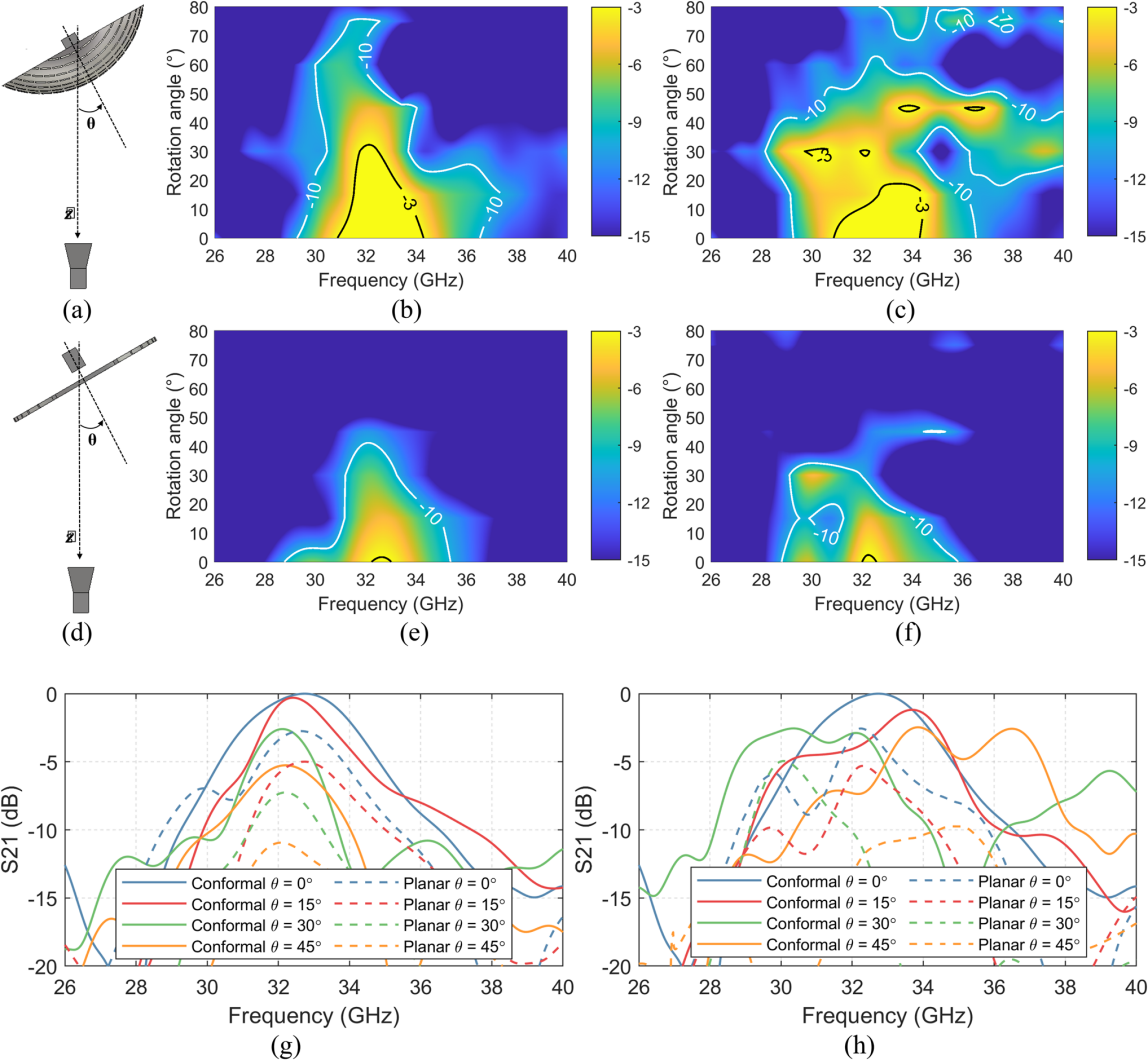
**(a)** Set up of conformal FSSs for different rotation angles along XZ plane; **(b)** simulated S21 results with the TE polarized incidence of the conformal FSS; **(c)** simulated S21 results with the TM polarized incidence of the conformal FSS; **(d)** set up of planar FSS for different rotation angles along XZ plane; **(e)** simulated S21 results with the TE polarized incidence of the planar FSS; **(f)** simulated S21 results with the TM polarized incidence of the planar FSS; **(g)** comparison between selective simulated S21 results of conformal FSS and planar FSS with the TE polarized incidence; and **(h)** with the TM polarized incidence;

One of the advantages of the conformal FSS, when compared to a planar FSS, is that the conformal FSS offers a larger angle of coverage when the incident wave angle changes. This behavior is simulated by rotating both the FSS and the OEWG in the *XZ* plane (varying *θ*), which is equivalent to having the horn antenna rotated along the axis of the center of the FSS – shown in Fig. 5(a) and (d). The distances *d* is chosen to ensure the passband center frequency and bandwidth of the two FSSs at *θ* = 0° being close to the infinite planar FSS array in the far-field, i.e. *d*_conformal_ = -5 mm and *d*_planar_ = 10 mm.

The simulated transmission results of the conformal FSS as the function of rotation angle and frequency in TE and TM polarizations are shown in Fig. 5(b) and (c), respectively; while the results of the planar FSS in TE and TM polarizations are shown in Fig. 5(e) and (f), respectively. The comparison between the conformal FSS and planar FSS with different polarization incidence are shown in Fig. 5(g) and (h). It is observed that the curved FSS offers better angular stability than the planar FSS in both TE and TM modes. With the TE polarized incidence, the conformal FSS keeps a − 3 dB bandwidth with the incidence angle up to 30º. The fields of view in TM mode of the conformal FSS is slightly narrower than the TE mode, with fields of view for a − 3 dB bandwidth up to 20º. By contrast, the planar FSS only keeps a steady -3 dB passband bandwidth up to ~ 5º in both TE and TM polarizations. Both the − 3 dB and − 5 dB passband bandwidth of the conformal FSS in TE mode is gradually reduced with the incidence angle increased, while the − 5 dB bandwidth in the TM mode keeps a similar level up to 40º. This transmission behavior of the conformal FSS with the increased incidence angle in both TE and TM mode is similar to the infinite planar FSS array in the far-field.

## Manufacturing

The process of manufacturing and metalizing this type of 3D structure was not a simple task. Many prototypes have been attempted to verify the printing parameters including support properties, plastic retraction, fan cooling, FSS orientation, and plate heating, which provide accurate FSSs dimensions and smooth surface. The manufacturing was carried out using Ultimaker 2 + . It worth noting that the overhanging parts caused by the conformal dome shape of the FSS were prone to deformation, which affected the FSS performance. Therefore, support structures were strategically chosen to ensure the overhanging parts of the FSS being fabricated correctly. Parameters including the support spacing, the distance between the supports and the main printed structure, support density, cooling speed were adjusted to provide the balance between good support yet could be easily removed, so that no remains stuck on the FSS and thus surface smoothness was improved. Furthermore, the supports density was locally tailored so that the supports did not affect the dimensions of the FSS slots.

**Figure. 6:**
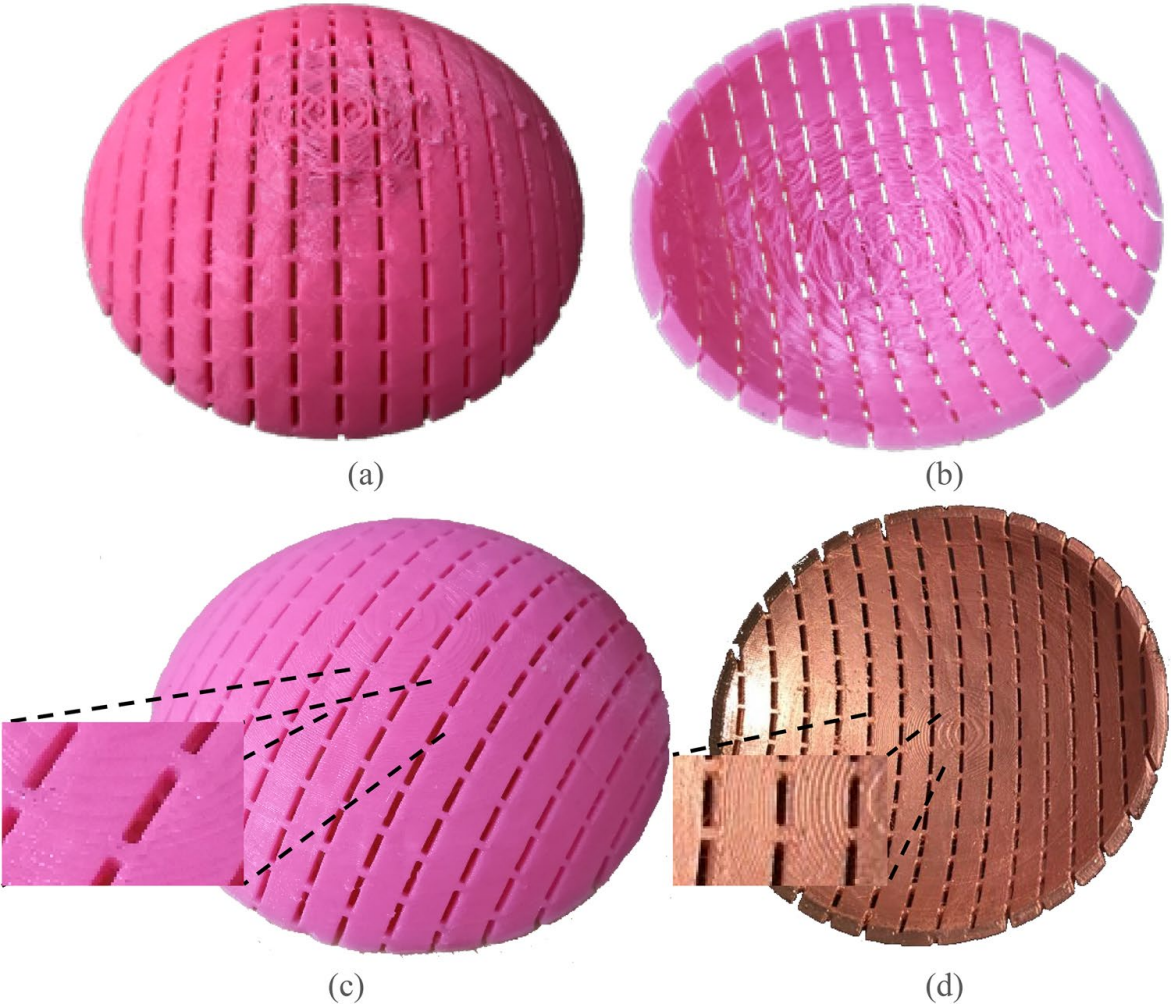
**(a)** Photo of the convex side of the 3D printed conformal FSS without using support structures when it was printed with the convex side face down; **(b)** photo of the concave side of the 3D printed conformal FSS without using support structures when it was printed with the convex side face up; **(c)** photo of the convex side of the 3D printed FSS with strategically selected support structures and optimized print settings when it was printed with the convex side face up; **(d)** photo of the 3D printed conformal FSS with the optimized print settings after metallization.

Two imperfect manufactured samples are presented in Fig. 6(a) and (b). Figure 6(a) corresponds to printing the structure with the convex face down, showing that the top face could not be properly filled. On the other hand, when the structure was printed face up as a dome but with no support, it resulted in the poor surface texture of the inner convex face (see Fig. 6(b)).

After several adjustments to the printing parameters and FSS arrangements, it was concluded that the best results were obtained when printing the FSS face up using support structures, whose parameters have been meticulously adjusted (see Fig. 6(c)). Some of the most critical parameters for the proposed FSS were: layer height = 0.1 mm, material infill = 10%, support enabled, support infill = 15%, support *z* distance = 0.15 mm, towers enabled, tower diameter = 2 mm, heat plate = 40 ºC, nozzle temperature = 210 ºC, fan speed = 100%, initial fan speed = 0%, regular fan speed at layer = 10. By using the optimal parameters, a smooth surface was obtained as shown in Fig. 6(c). Following the 3D printing of the FSS in PLA plastic, the parts were then metalized using copper electroplating to produce the copper FSS shown in Fig. 6(d).

**Figure. 7:**
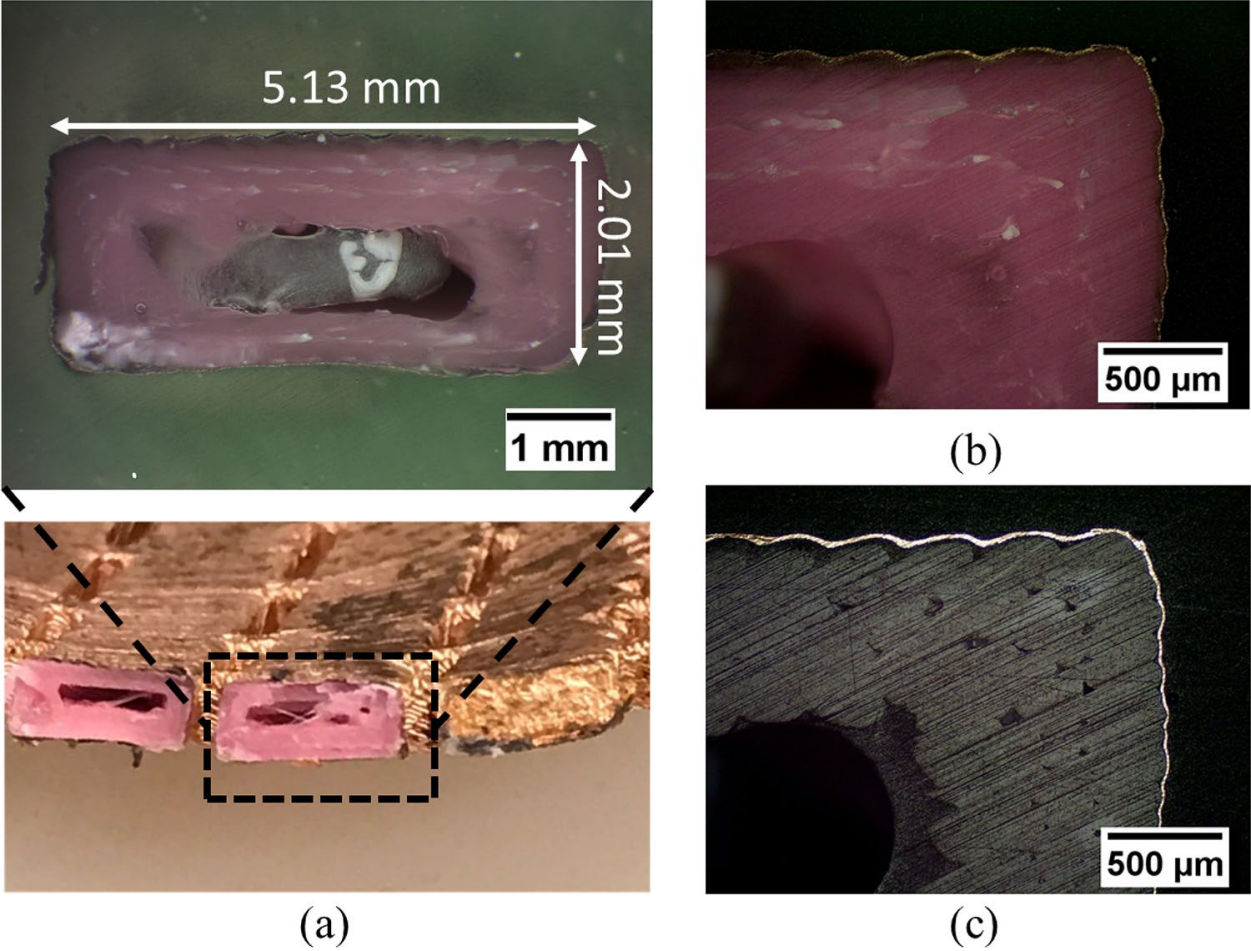
**(a)** Cross-sectional area of the FSS after copper plating, **(b)** zoomed view under microscope, and **(c)** high contrast view to illuminate the metallization layer.

**Figure. 8:**
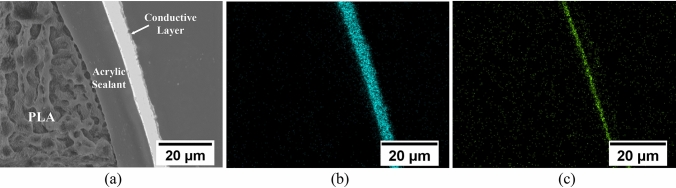
**(a)** SEM micrographs of the FSS after the copper plating; **(b)** energy-dispersive X-ray spectroscopy (EDS) element maps for raw materials copper and **(c)** silver distribution.

The first step of the metallization was to coat the parts with an acrylic sealant to reduce any porosity resulting from micro-gaps between the 3D printed layers. Once dry, a seed conducting layer was applied to the sealed plastic using an airbrush. The seed layer was a high-grade silver-based conductive ink from Spa Plating and provides an initial conductive layer upon which electroplating of copper could be implemented. The ink was cured at room temperature for more than 24 h. The FSS was then placed in crocodile clips connected to a power source and immersed in a copper sulphate bath as the cathode. The surface area (two sides) of an FSS was approximated to be 126 cm^2^, and a target copper thickness of 5 microns was used. Initial plating was done at a modestly low current (~ 0.2 A) to ensure good copper deposition across the whole of the FSS; too high an initial current could lead to localized high copper deposition or burnt deposits on the FSS around the crocodile clips. Once the FSS was observed to have a copper layer deposit encapsulating it, then the current was increased (~ 1 A) to accelerate the rate of copper deposition. After the plating process, the FSS was rinsed thoroughly with deionized water. The cross-section of the FSS after being copper plated was examined under an optical microscope, shown in Fig. 7(a). The polished cross-section was taken at the long spacer area between two slots. It shows that the space between the slots (~ 5.13 mm) agrees with the designed value. It also shows the hollow structure that benefits the mass reduction for the FSS. Figure 7(b) and (c) clearly show the deposited metallization layer. To further investigate the metallization quality, a Scanning Electron Microscope (SEM) image was taken – shown in Fig. 8(a), which reveals an acrylic sealant layer and a conductive layer with average thicknesses of 15 microns and 6 microns, respectively. The Energy-Dispersive X-ray Spectroscopy (EDS) element maps for copper and silver are shown in Fig. 8(b) and (c). It confirms that the whole conductive layer is composed of a silver seed layer (thickness of ~ 1 micron) that is attached to the acrylic sealant layer and a copper layer (thickness of ~ 5 microns).

### Characterization of conformal FSS

**Figure. 9:**
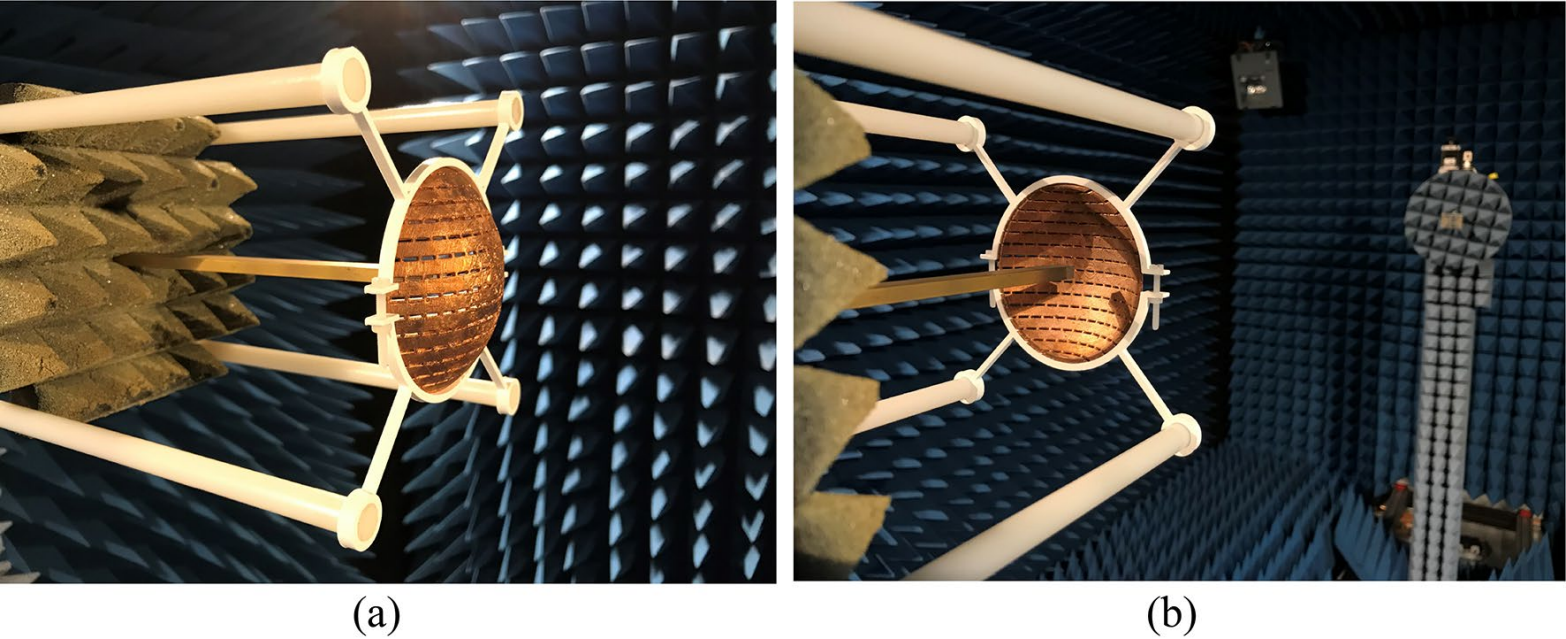
Measurement setup for the spherical dome FSS **(a)** the OEWG is placed *inside* the FSS, **(b)** view from the Rx to the Tx.

The measurement set-up was similar to the one used in the simulation. A WR-28 open-ended waveguide and a double ridged horn antenna were used as the receiver and as the transmitter, respectively. The measurements were conducted in the anechoic chamber (see Fig. 9).

**Figure. 10:**
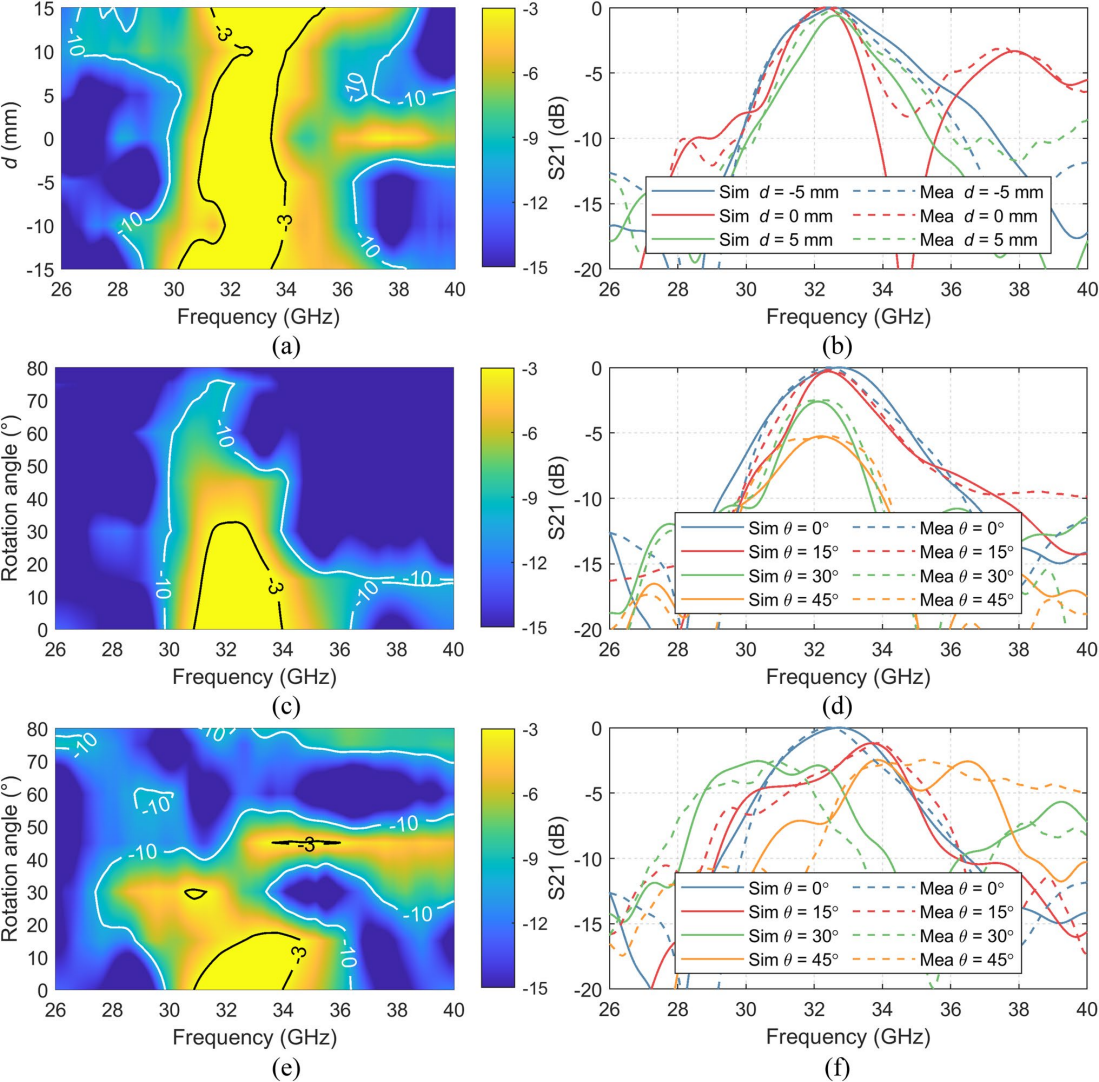
**(a)** Colormap of measured S21 of the conformal FSS as a function of the distance between the FSS and the receiver; **(b)** comparison between selective simulated and measured S21 results as a function of the distance; **(c)** colormap of measured S21 of the conformal FSS as a function of the angle in TE incidence; **(d)** comparison between selective simulated and measured S21 results as a function of the angle in TE incidence; **(e)** colormap of measured S21 of the conformal FSS as a function of the angle in TM incidence; **(f)** comparison between selective simulated and measured S21 results as a function of the angle in TM incidence.

The measurement results of the curved FSS for different positions of the receiver are presented in Fig. 10. Comparing both the simulation (Fig. 2(b)) and measurement results (Fig. 10(a)), excellent agreement between the simulated and measured data is observed. The slight discrepancies were mainly due to the difficulty of placing the OEWG at exactly the required distance from the FSS, since a 3D printed support was used as one can see in Fig. 10(a) and (b), which had certain tolerances. This could be improved by utilizing more precise support. Another source of error was the approximated solution that was used in the simulation due to RAM restriction. Regardless of the minor difference between simulation and measurement, however, similar conclusions to the ones obtained from simulations could be extracted: the conformal FSS offers similar − 3 dB bandpass bandwidth of approximately 11% with a stable center frequency of around 32.5 GHz. Moreover, when the OEWG was “inside” the FSS, its bandpass behavior was less changeable than when it was outside, i.e. larger − 5 dB bandwidth was observed when the OEWG was inside the FSS, since a similar range of incidence angles impinges on the FSS. On the other hand, a larger variation on the passband properties was observed when the OEWG was “outside”, owing to the same reasons specified above. Therefore, it confirmed that the behavior of the structure when the OEWG was placed “outside”, was less controllable.

The measured S21 results of the conformal FSS with different incidence angles when excited with both TE and TM polarized waves are shown in Fig. 10(c) and (d), which demonstrates the advantage of the wide coverage of the conformal FSS. The measured transmission responses well agree with the simulated data (simulated TE mode in Fig. 5(b) and TM mode in Fig. 5(c)). The measured -3 dB passband properties of the curved FSS are kept up to 30º and − 5 dB bandwidth was up to 45º with a steady center frequency around 32.5 GHz when excited with the TE polarized wave. When it is illuminated by the TM wave, the − 3 dB and − 5 dB passband is kept up to 20º and 30º, respectively. But the passband center frequency of the FSS in the TM mode is less stable compared with the TE mode. An improved unit cell design with a better symmetrical structure can enhance the angular stability in TM mode.

**Figure. 11:**
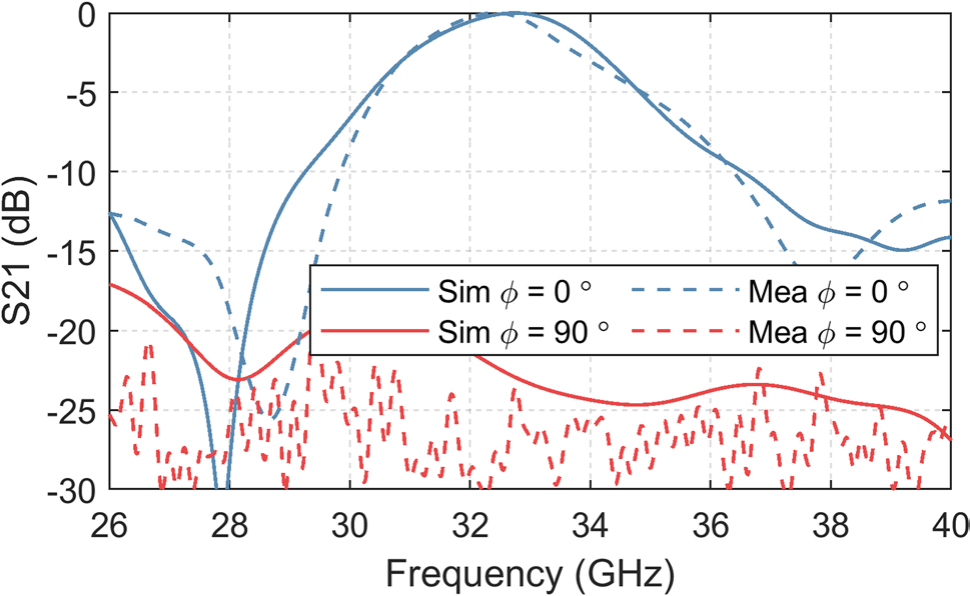
Rejection properties of the conformal FSS, measured by rotating it in the XY plane.

The rejection capabilities of the conformal FSS are analyzed when rotating only the FSS in the XY plane, keeping the horn antennas orientation (cross-polarization analysis in analogy to the antenna measurements). The curved FSS was rotated by *ϕ* = 90º for comparing with the transmission when *ϕ* = 0º. When *ϕ* = 90º the incident magnetic field was perpendicular to the slot orientation and hence, no electromagnetic resonance took place on the structure. Consequently, it was similar to a continuous metallic structure and the incident electromagnetic wave was reflected. The measured S21 results of the conformal FSS with the co-polarization and cross-polarization incidences are shown in Fig. 11, compared with simulated results. The simulation and measurement results show up to ~ -30 dB cross-polarization level within the passband frequency range. Furthermore, the conformal FSS provides a good shielding ability for the cross-polarization incident waves, with S21 of ~ -25 dB in the entire frequency range from 26 to 40 GHz.

## Conclusions

The concept of using the conformal FSS in the near-field region is introduced in this paper, aiming to provide improved bandpass performance compared to the equivalent finite planar FSS structure. A Ka-band spherical dome FSS array was 3D printed on PLA, metalized, and validated the bandpass performance and fabrication technique. The simulation and measurement results have shown to be in close agreement. The passband properties of the structure were presented showing that the use of a conformal FSS provides better angular stability and is less sensitive to the distance between the FSS and the receiver, compared to the finite planar case.

The chosen spherical dome shape is to support the quasi-spherical wavefront from the OEWG. The simple shape means that it can be fabricated in-house using the low-cost FFF 3D printer. More exotic shapes are possible to be realized but require more complicated supporting structures. In this work, the challenge of deploying the FSS on the conformal surface without FSS element distortion has been addressed by using the AM technique. Further developments could be made with the utilization of improved resolution of 3D printed elements with a variation of geometries. Furthermore, AM gives the freedom of allowing a mixture of elements (e.g. slots, cross slots, etc.) with different dimensions on the curved array from the centre of the surface to the edges, so the edge effect can be mitigated.

This new concept of curved FSS can be applied to build other types of metasurfaces, such as transmitarrays, lens, etc. and could be used as a tunable polarizer for widening or narrowing its passband properties.

**Table 1:** Passband parameters of the conformal FSS.

*D* (mm)	f_min_ (GHz)	f_c_ (GHz)	f_max_ (GHz)	BW (GHz)	BW (%)
− 15	30.8	31.9	33.6	2.8	8.9
− 10	30.5	32.4	33.8	3.3	10.2
− 5	30.9	32.6	34.4	3.6	11.0
0	31.1	32.3	33.2	2.1	6.6
5	31.7	32.6	33.6	1.9	5.9
10	32.3	33.0	33.7	1.3	4.0
15	31.1	32.4	35.5	4.4	13.6
Infinite planar FSS in far-field	30.9	32.6	34.3	3.3	10.2

**Table 2:** Passband parameters of the planar FSS.

*D* (mm)	f_min_ (GHz)	f_c_ (GHz)	f_max_ (GHz)	BW (GHz)	BW (%)
5	32.1	32.9	33.7	1.6	4.9
10	31.6	32.7	33.7	2.1	6.5
15	32.0	32.8	33.6	1.6	4.7
20	NaN	32.7	NaN	NaN	NaN
Infinite planar FSS in far-field	30.9	32.6	34.3	3.3	10.2
